# Evidence for long noncoding RNA GAS5 up-regulationin patients with Klinefelter syndrome

**DOI:** 10.1186/s12881-018-0744-0

**Published:** 2019-01-07

**Authors:** Michele Salemi, Rossella Cannarella, Rosita A. Condorelli, Laura Cimino, Federico Ridolfo, Giorgio Giurato, Corrado Romano, Sandro La Vignera, Aldo E. Calogero

**Affiliations:** 1 Oasi Research Institute-IRCCS, Troina (EN), Italy; 2 Department of Clinical and Experimental Medicine, University of Catania, Catania, Italy; 3 UOS of Clinical Pathology, ASUR Marche – AV2, Hospital of Senigallia, Senigallia, Italy; 4 Genomix4Life Srl, Department of Medicine, Surgery and Dentistry “Scuola Medica Salernitana”, University of Salerno, Baronissi (SA), Italy

**Keywords:** Klinefelter syndrome, Rare disease, Cognitive deficits;NGS, mRNA, qRT-PCR

## Abstract

**Background:**

Klinefelter syndrome (KS) is characterized by the presence of at least one supernumerary X chromosome. KS typical symptoms include tall stature, gynecomastia, hypogonadism and azoospermia. KS patients show a higher risk of developing metabolic and cardiovascular diseases, inflammatory and autoimmune disorders, osteoporosis and cancer. Long non-coding RNA (lncRNA) growth arrest-specific 5 (*GAS5*) has been shown to be involved in several biologic processes, including inflammatory and autoimmune diseases, vascular endothelial cells apoptosis and atherosclerosis, as well as cellular growth and proliferation, cellular development and cell-to-cell signaling and interaction. The lncRNA GAS5 expression profile in KS patients has never been evaluated so far.

**Methods:**

To accomplish this, GAS5 mRNA levels were evaluated by Next Generation Sequencing (NGS) technology and qRT-PCR assay in 10 patients with KS and 10 age-matched controls.

**Results:**

NGS results showed a significantly lncRNAGAS5up-regulation by 5.171-fold in patients with KS. Theresults of qRT-PCR confirmed the NGS data.

**Conclusions:**

These findings showed the occurrence of lncRNA GAS5 over-expression in KS patients. Whether this lncRNA is involved in the pathogenesis of inflammation and autoimmune diseases, atherogenesis or germ cell depletion in KS patients is not known. Further studies are needed.

## Background

Klinefelter syndrome (KS) is the most common sex-chromosome disorder in men, with an estimated prevalence of 1:660 newborns [1, 2]. The most common karyotype is the classic 47,XXY one, which accounts for the 80–90% of all cases [3]. It is a consequence of a non-disjunction of paired X-chromosomes during the first or second meiotic division [3], equally due to a paternal or maternal meiotic mal segregation event [4]. The remaining 10% of KS are due to chromosome mosaicisms (e.g. 46,XY/47,XXY) or to more complex karyotypes (X chromosome structural abnormalities such as 47,XX, der(Y), 47,X, der(X),Y, or other numeric sex chromosome abnormalities such as 48, XXXY, 48, XXYY and 49, XXXXY) [3, 5].

The chromosomal abnormality leads to a progressive germ cell degeneration starting from mid-puberty, impaired Sertoli cell function [6], total tubular atrophy or hyalinizing fibrosis and Leydig cell hyperplasia [7], clinically causing hypergonadotropic hypogonadism [8], small testis with increased consistency and infertility. Occasionally, foci of spermatogenesis have been observed in testis of KS patients [7]. Clinically, azoospermia is present in the 90% of non-mosaic KS, whereas severe oligozoospermia in the remaining 10% [7].

Several clinical manifestations associate with the syndrome. These include learning and language disability, reduction in intelligence quotient (IQ) scores of 10 to 15 points, but not into the intellectual disability range [6], increased risk for mitral valve prolapse, lower-extremity varicose veins, venous stasis ulcers, deep vein thrombosis and pulmonary embolism, autoimmune diseases, 20-fold-higher risk of developing breast cancer, type II diabetes mellitus (T2 DM) and metabolic syndrome [9], osteoporosis [10], extragonadal germ cell tumors and non-Hodgkin lymphoma [11, 12].

Molecular mechanisms underlying the variability in KS phenotype have not been clearly understood yet. Epigenetic mechanisms have been suggested to play a role [13]. Growth arrest-specific 5 (*GAS5*) gene, mapping on the 1q25.1 chromosome, encodes for a long non-coding RNA (lncRNA) which is involved in the modulation of gene expression, targeting many different downstream miRNAs [14, 15].

LncRNA GAS5 was initially identified as a tumor suppressor gene [16–21]. However, it has also been shown to support female germline stem cell (FGSC) survival [22] and adipocyte differentiation [15]. In addition, recent evidence pointed to lncRNA GAS5 a role in atherosclerosis [23] and autoimmune diseases [24], which are widely recognized as KS comorbidities.

No study evaluated the lncRNA GAS5 expression profile in KS patients so far. On this account, the present study was undertaken to investigate differently expressed genes and, specifically, lncRNA GAS5 expression in patients with KS and controls.

## Methods

The present study belongs to a broad project investigating transcriptome differences between patients with KS and normal control subjects (NC). To accomplish this, 10 patients with non-mosaic KS and 10 healthy controls of similar age were selected. All men with KS (mean age 32.5) had 47,XXY karyotype, as confirmed by cytogenetic investigation performed on at least 50 metaphases. NC (mean age 32) had negative history of genetic diseases, normal testis volume and normal reproductive hormones (LH, FSH, total testosterone) levels. All men enrolled in this study (KS patients and controls) were Italians.

They underwent to transcriptome and qRT-PCR analysis which were performed on peripheral blood mononuclear cells (PBMCs) isolated with ficoll gradient.

### RNA sequencing

RNA Sequencing, Next Generation Sequencing (NGS) and Data Analysis of NGS methods have been detailed in previous articles presenting NGS data from the same project [13, 25].

Indexed libraries were prepared from 1 μg/ea. purified RNA with TruSeq Stranded Total RNA (Illumina) Library Prep Kit according to the manufacturer’s instructions. Libraries were quantified using the Agilent 2100 Bioanalyzer (Agilent Technologies) and pooled such that each index-tagged sample was present in equimolar amounts, with final concentration of the pooled samples of 2bnM. The pooled samples underwent cluster generation and sequencing using an IlluminaHiSeq 2500 System (Illumina) in a 2 × 100 paired-end (RNA-seq) format. The raw sequence files generated (.fastq files) underwent quality control analysis using FastQC (https://www.bioinformatics.babraham.ac.uk/projects/fastqc/).

### Data analysis

Bioinformatics analysis were performed by Genomix4Life srl (Baronissi(SA), Italy). The quality checked reads were trimmed with cutadapt v.1.10 and then aligned to the human genome (hg19 assembly) using STAR v.2.5.2 [26], with standard parameters. Differentially expressed mRNAs were identified using DESeq2 v.1.12 [27].

Gene annotation, as provided by Ensembl (GRCh37), was obtained for all known genes in the human genome. We calculated the number of reads mapping to each transcript with HTSeq-count v.0.6.1 [28]. These raw read counts were then used as input to DESeq2 for calculation of normalized signal for each transcript in the samples, and differential expression was reported as fold change along with associated adjusted *p*-values (computed according to Benjamini-Hochberg). Differential expression data were further confirmed using Cuffdiff [28]. Functional analysis was performed using Ingenuity Pathway Analysis (IPA) with standard parameters.

### Availability of data and materials

Raw data are available in ArrayExpress database repository (https://www.ebi.ac.uk/arrayexpress/experiments/E-MTAB-6107/) with accession number E-MTAB-6107.

### Validation whit qRT-PCR

To validate the results obtained by NGS analysis, we compared RT-PCR in 10 KS patients and 10 NC. Retro-transcription was performed with QuantiTect Reverse Transcription Kit (QIAGEN Sciences, Germantown, PA). Briefly, 600 ng of total RNA from each sample was then reconstituted in a final volume of 20 μl and the generated cDNA was used as a template for real-time quantitative PCR analysis using gene expression products. Quantification with qRT-PCR was performed using the 2^-ΔΔCt^ method [29] by matching each KS patient to a respective NC aged ±3 years.

The target *GAS5* and the reference gene glyceraldehyde-3-phosphate dehydrogenase (*GAPDH*) TaqMan assays (Assay ID:Hs99999905_m1) were obtained from Applied Biosystems (Carlsbad,CA, USA).

The *GAS5* TaqMan assays (Assay ID: Hs03464472_m1) used amplifies an amplicon between exon 10 and exon 11 (it is possible to check the characteristics of the probe used at the following link: https://www.thermofisher.com/taqman-gene-expression/product/Hs03464472_m1?CID = &ICID = &subtype=). The mean was obtained with the Software Version 1.5 supplied with the LightCycler 480, as previously reported [30].

Distribution analysis of measured gene transcript levels was performed using Shapiro – Wilk test and inferential statistical analysis of results was carried out using paired two tailed *t*-test and bivariate linear regression analysis. Graph Pad Prism 5 software was used for statistical analysis. A *p* value < 0.05 was accepted as significant.

## Results

**Fig. 1:**
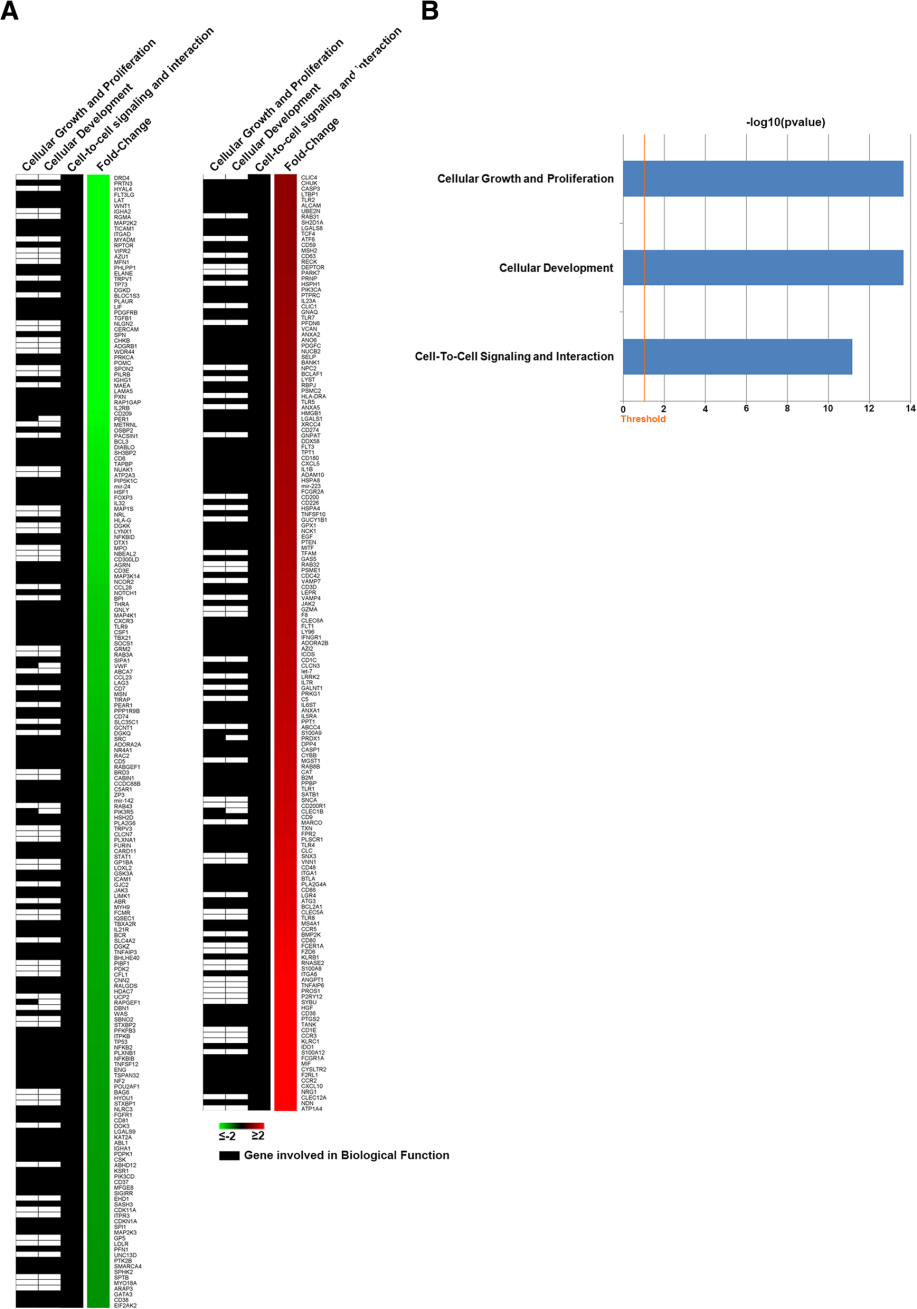
Heatmap of gene functions. **a**. Heatmap showing the differentially expressed genes (|FC| ≥ 2 and p-adj ≤ 0.05) in whose function GAS5 is involved. **b**. Histogram showing the statistical significance of the functions where GAS5 is involved. The length of the bar is inversely proportional to the *p*-value

Clinical features and biochemical data of KS patients and controls have been reported elsewhere [13, 25]. Differential expression analysis showed 4448 genes differentially expressed with |fold-change| ≥2 and p-adj ≤ 0.05 comparing KS patients versus the 10 controls. Among these, 2698 were down-regulated and 1750 were up-regulated and Ingenuity Pathway Analysis (IPA) highlighted their involvement in several biological functions, including cellular growth and proliferation, cellular development and cell-to-cell signaling and interaction, where the transcription of *GAS5* (locus 1:173832385–173,872,687) was up-regulated in the patients with KS by 5.171-fold (q-value< 0.0001) (Fig. 1).

qRT-PCR was performed to validate these findings. Accordingly, the high sensitivity of this assay is optimal for ensuring accuracy in the evaluation of gene transcription levels. We examined lncRNA levels of *GAS5* in 10 KS patients and 10 age-matched controls.

**Fig. 2:**
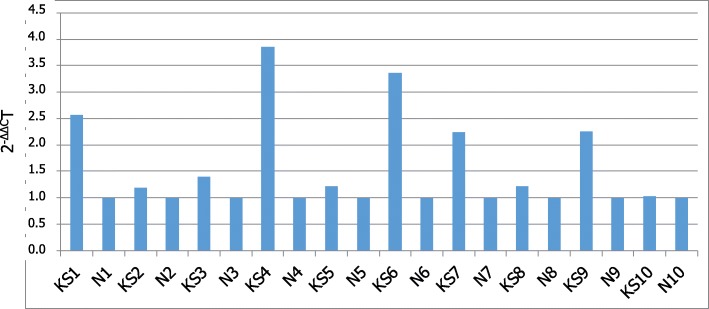
*GAS5* lncRNA expression in men with Klinefelter syndrome and normal controls. Data obtained by qRT-PCR. N, controls

**Fig. 3:**
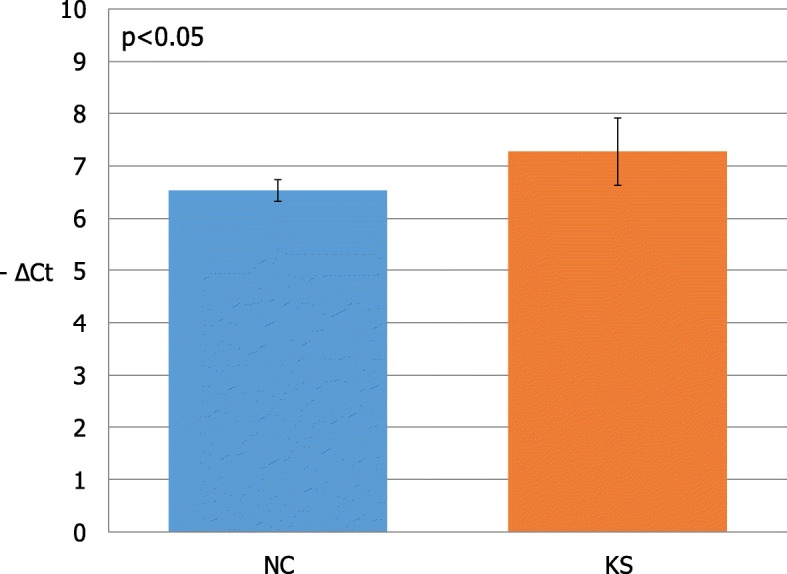
Mean of lncRNA GAS5 expression values in patients with Klinefelter syndrome and normal controls. KS, Klinefelter syndrome; NC, normal controls

Distribution of expression values was normal in both KS patients and NC (*p* > 0.01). Therefore, we carried out a statistical inferential analysis using the parametric test paired two tailed *t*-test. Data acquired from this analysis revealed a significant increase of *GAS5*mRNA levels in KS patients compared to relative controls (*p* < 0.01), as shown in Fig. 2. In addition, we investigated the expression of the two groups by evaluating the relative expression value in terms of -ΔCt (Fig. 3). In this analysis, the mean expression of NC was 6.53 (value range 6.31–6.8; SD = 0.2; CV = 0.02; IC 95% 6.21–6.85), whereas the mean expression of the KS subjects was 7.28 (value range 6.55–8.28; SD = 0.65; CV = 0.085; IC 95% 6.81–7.75). Inferential statistical analysis reveals significant difference between two means expression value (*p* < 0.05). Linear regression analysis reveals no significant correlation between GAS5 mRNA levels and age of subjects involved in our study (*p* > 0.05).

Table 1 shows the main NGS findings validated by qRT-PCR presented so far that belong to the same project [13, 25]. The results confirm the data obtained by the NGS analysis and differences in values reflect the diversity of methods.

**Table 1:** Differently-expressed transcripts in patients with Klinefelter syndrome (KS) and controls

	KS patients vs. controls	Reference
MIR3648	Down-regulated	Cimino et al., 2017 [13]
MIR3687	Down-regulated	Cimino et al., 2017 [13]
MT-ND6	Down-regulated	Salemi et al., 2018 [25]
LncRNA GAS5	Up-regulated	Present study

Finally, fitting with the suppressing role of lncRNA GAS5 on IGF1R expression [31], our NGS data show that the IGF1R gene is down-regulated in KS subjects (FC = − 1.873 and p-adj = 0.000126) compared to normal subjects.

## Discussion

We recently reported a decreased miRNAs and mitochondrial subunits expression in KS patients compared to controls [13, 25]. In the present study, we showed, for the first time, the occurrence of a higher lncRNA GAS5 expression in patients with KS compared to controls.

LncRNA GAS5 is involved in several biologic processes, including glucocorticoid (GC) actions, inflammatory and autoimmune diseases, vascular endothelial cells apoptosis and atherosclerosis, FGSC proliferation.

LncRNA GAS5 could directly interact with the DNA binding domain of the glucocorticoid receptor (GR) [32]. This prevents receptor binding to the GC responsive element (GRE) in target genes, blocking their transcription [32]. Because of its competition with GRE, lncRNA GAS5 is considered to act as a repressor of the GR action [33]. Accordingly, poor responders to GCs showed higher lncRNA GAS5 levels compared to normal ones [34]. GCs have a regulatory action on the immune system and are employed in inflammatory and autoimmune diseases. Altered lncRNA GAS5 levels in whole blood or leucocytes of patients with rheumatoid arthritis, systemic lupus erythematosus, multiple sclerosis, sarcoidosis, osteoarthritis and inflammatory bowel disease have already been reported [24, 35, 36], thus suggesting that lncRNA GAS5 may play a role in the pathogenesis of inflammatory and autoimmune diseases (probably modulating responsiveness to endogenous GCs), which are more frequent in KS patients.

LncRNA GAS5 expression is involved in atherogenesis. Its expression is significantly higher in plaques of atherosclerosis both from human and from animal models. Its over-expression enhances vascular endothelial cells apoptosis after lipoperoxidation. Therefore, lncRNA GAS5 has been suggested as a target for the therapy of atherosclerosis [23]. KS patients have an increased risk of atherogenesis, due to hypogonadism and metabolic syndrome [37]. Since the aforementioned evidence, a role for lncRNA GAS5 in atherogenesis in KS patients cannot be excluded.

Due to the involvement of lncGAS5 in several biological functions required for normal spermatogenesis, such as cellular growth and proliferation, cellular development and cell-to-cell signaling and interaction (Fig. 1), we speculate that lncGAS5 deregulation may play a role in germ cell loss in KS patients.

Very recently, lncRNA GAS5 has been regarded as a promoter of proliferation and survival of FGSC [22]. In greater detail, it has been found highly expressed in FGSCs and oocytes from neonatal mice and to facilitate cultured FGSC survival through the inhibition of their apoptosis in-vitro [22]. No study explored lncRNA GAS5 expression in male germ cells so far. This knowledge may help to understand its role, if any, in germ cell loss in KS patients. Indeed, lncRNA GAS5 has been shown to down-regulate the *IGF1R* expression [31]. Accordingly, we found that *IGF1R* expression was down-regulated in KS patients compared to controls. Growing evidence addressed to *IGF1R* a role in human testicular function [38] and in Sertoli cell proliferation and function [39]. Indeed, its knock-out (both the single one and in combination with the *Insr*) in Sertoli cells from mice affected the final testicular size, Sertoli cell number and the sperm output [40]. Therefore, a down-regulation of *IGF1R* in Sertoli cells might hypothetically explain germ cell loss in KS patients.

Finally, apoptosis is a mechanism responsible for the normal regulation of germ-cell death during differentiation and maturation of normal human germ cells. It is a prerequisite for a normal spermatogenesis [41, 42]. Therefore, apoptosis may contribute to the excessive germ-cell demise in KS patients. According with Koldemir and colleagues (2017), GAS5 accumulation in exosomes during apoptotic induction promotes two different mechanisms, microtubule stabilization and DNA strand breaks [43]. Therefore, it is plausible to hypothesize that GAS5 is involved in communication of cells upon cell death-promoting signals.

## Conclusions

In conclusion, the results of this study showed a significant over-expression of lncRNA GAS5 in patients with non-mosaic KS compared to controls. The lncRNA seems to play a role in various biologic processes, such as GC actions, inflammatory and autoimmune diseases, vascular endothelial cells apoptosis and atherosclerosis and in FGSC proliferation. Furthermore, its involvement in cellular growth and proliferation, cellular development and cell-to-cell signaling and interaction suggests a possible role in germ cell loss. However, whether the lncRNA GAS5 over-expression is really involved in the pathogenesis of some of the typical features of KS patients (particularly in inflammatory and autoimmune diseases, atherogenesis and germ cell loss) needs to be further investigated.

## Abbreviations

FGSC: Female germline stem cell
GAS5: Growth arrest-specific 5
GC: Glucocorticoid
GR: Glucocorticoid receptor
GRE: Glucocorticoid responsive element
IQ: Intelligence quotient
KS: Klinefelter syndrome
lncRNA: Long non-coding RNA
NGS: Next Generation Sequencing
PBMCs: Peripheral blood mononuclear cells
T2DM: Type II diabetes mellitus
